# Test and treat COVID 65 plus - Hydroxychloroquine versus placebo in early ambulatory diagnosis and treatment of older patients with COVID19: A structured summary of a study protocol for a randomised controlled trial

**DOI:** 10.1186/s13063-020-04556-z

**Published:** 2020-07-10

**Authors:** Siri Göpel, Wolfgang Bethge, Peter Martus, Florian Kreth, Thomas Iftner, Stefanie Joos, Stefanie Döbele, Benjamin Mordmüller, Peter Kremsner, Thomas Ettrich, Thomas Seufferlein, Michael Bitzer, Nisar Malek

**Affiliations:** 1 Department of Internal Medicine 1, University Hospital Tübingen, Tübingen, Germany; 2 Department Internal Medicine 2, University Hospital Tübingen, Tübingen, Germany; 3 Institute for Clinical Epidemiology and Applied Biostatistics, University of Tübingen, Tübingen, Germany; 4 Department of Virology, University Hospital Tübingen, Tübingen, Germany; 5 Department of General Medicine, University Hospital Tübingen, Tübingen, Germany; 6 Institute for Tropical Medicine, University Hospital Tübingen, Tübingen, Germany; 7 Medical Clinic 1, University Hospital Ulm, Ulm, Germany

**Keywords:** COVID-19, Randomised controlled trial, Protocol, Early treatment, Elderly patients, Hydroxychloroquine, Placebo-controlled, Double-blind, Ambulatory, Outpatient, Moderate disease

## Abstract

**Objectives:**

The aim of this trial is to identify the effect of ambulatory treatment in early COVID-19 disease with hydroxychloroquine on the rate of hospitalization or death in older patients above the age of 64.

**Trial design:**

Parallel, 2:1 randomization, double blind, placebo-controlled, multi-center trial.

**Participants:**

Male and female patients above the age of 64 (i.e. ≥65 years of age) with COVID-19 diagnosis confirmed by SARS-CoV2 positive throat swab (PCR). Patients can only be included within 3 days of symptom onset in ambulatory care if they consent to the study procedure and are able to adhere to the study visit schedule and protocol requirements (including telephone visits concerning symptoms and side effects). Severity of disease at inclusion is mild to moderate defined as not requiring hospital admission: SpO2 >94%, respiratory rate <20, mental state alert, no signs of septic shock. Cardiac risk is minimised by requiring a Tisdale score ≤ 6. Patients are recruited in the two german cities of Ulm and Tübingen in various ambulatory care settings.

**Intervention and comparator:**

Each patient will be given a first dose of 600 mg Hydroxychloroquine or the equivalent number of placebo capsules (3 capsules) at the day of inclusion. From the 2^nd^ day on, each patient will get 200 mg or the equivalent number of placebo capsules twice a day (400mg/day) until day 7 (6 more does of 400 mg); a cumulative dose of 3 g.

**Main outcomes:**

Rate of hospitalization or death at day 7 after study inclusion

**Randomisation:**

All consenting adult patients having confirmed COVID-19 are randomly and blindly allocated in a 2:1 ratio to either IMP or placebo. The biostatistical center produced a randomization list (block randomization) with varying block length and stratified for the study center. This list is provided for packaging to the pharmaceutical unit which is providing encapsulated placebo and IMP. Only the pharmaceutical unit is aware of group allocation according to the randomization list.

**Blinding (masking):**

Patients and investigators, as well as treating physicians are blinded to the treatment- allocation.

**Numbers to be randomised (sample size):**

In the first stage of an adaptive design 120 patients in a 2:1 ration: 72 Verum and 36 Placebo, plus an increase for 10% drop outs. After interim analysis, the total sample size will be calculated based on the effect seen in the first stage. Total sample size is estimated approximately n = 300-400 patients.

**Trial Status:**

Protocol version number: V3, 19.05.2020 Recruitment not yet started but is anticipated to begin by June 2020 and be complete by December 2020

**Trial registration:**

ClinicalTrials.gov: NCT04351516, date: 17 April 2020 EudraCT: 2020-001482-37, date: 30 March 2020

**Full protocol:**

The full protocol is attached as an additional file, accessible from the Trials website (Additional file 1). In the interest in expediting dissemination of this material, the familiar formatting has been eliminated; this Letter serves as a summary of the key elements of the full protocol.

## Supplementary information

**Additional file 1.** Full Study Protocol.

## Data Availability

Study results will be made available to the public by presentations at scientific meetings and by publication in a scientific journal.

