# Supplementary material for: Test and treat COVID 65 plus - Hydroxychloroquine versus placebo in early ambulatory diagnosis and treatment of older patients with COVID19: A structured summary of a study protocol for a randomised controlled trial
Source: Trials. 2020 Jul 10;21:635. doi: 10.1186/s13063-020-04556-z (PMC7348125; doi:10.1186/s13063-020-04556-z)
Supplement: Supplementary file 1 — Additional file 1. Full Study Protocol. [file 13063_2020_4556_MOESM1_ESM.pdf]

Randomized controlled trial of hydroxychloroquine versus placebo in early ambulatory diagnosis and treatment of elderly COVID19 Patients

Study code	COVID 65 plus
Short Title of Clinical Trial	Test and Treat COVID 65plus
Protocol Version	V3.0
Date of Protocol	18.05.2020
EudraCT-Number	2020-001482-37
Phase	II/III
Sponsor	Universitätsklinikum Tübingen,<br>Ärztlicher Direktor: M. Bamberg<br>Kaufmännische Direktorin: G. Sonntag,<br>Geissweg 3<br>72076 Tübingen<br>Germany
Investigational Medicinal Product	Hydroxychloroquine sulfate
Comparator	Placebo
Summary of the revision history (amendments)	Substitute Version 2 15.4.2020

II. Table of Contents

I. Title Page 1
II. Table of Contents 2
II.a) List of Tables 4
II.b) List of Figures 4
III. Signature Page 5
IV. Contacts 7
V. Abbreviations 9
VI. Synopsis 10
1. Introduction 15
1.1. Trial Rationale / Justification 15
1.2. Benefit / Risk Assessment 17
1.3. Advisory Committee 18
Data and Safety Monitoring Board (DSMB): 18
2. Study Objectives 19
2.1. Primary Objective and Endpoint 19
2.2. Exploratory Objectives and Endpoints 19
3. Study Design 20
3.1. Study Duration and Schedule 20
3.2. End of Study 21
4. Study Population 22
4.1. General Criteria for Subject Selection 22
4.1.1. Inclusion Criteria 22
4.1.2. Exclusion Criteria 22
5. Information on the Investigational Medical Product 24
5.1. Manufacturing of the Investigational Medicinal Product 24
5.2. Labeling of the Investigational Medicinal Product 24
5.3. Storage of the Investigational Medicinal Product 24
5.4. Drug Accountability, Therapy Compliance and Disposal 24
5.5. Method of Treatment Assignment 25
5.6. Dose Schedule 25
6. Study Procedures and Examination Method 26
6.1. Study Entry 26
6.1.1. Patient's Informed Consent 26
6.1.2. Screening and Enrollment 26
6.1.3. Randomisation 26
6.1.4. Blinding 27
6.1.4.1. Unblinding 27
6.1.5. Concomitant Medication and Treatments 27
6.2. Treatment Phase 27
6.3. Assessment of Efficacy 29
6.4. Assessment of Safety 29
6.5. Premature termination of clinical trial for a trial subject 29
6.6. Premature closure of a trial site 30
6.7. Premature termination of the trial 31
6.8. Follow Up 31
6.9. End of Study for Subjects 31
6.9.1. Plan for Treatment or Care after End of Study 31
7. Quality control and Quality assurance 32

7.1. Risk-based approach 32
7.2. Monitoring 32
7.3. Audits/ Inspections 33
7.4. Documentation: Collection, Handling, Storage and Archiving of Data 33
7.4.1. Case Report Form 33
7.4.2. Source Data 33
7.4.3. Data Handling 34
7.4.4. Storage and Archiving of Data 34
8. Statistical Analyses 35
8.1. Study Population Definition 35
8.1.1. Sample Size and Power Consideration 35
8.2. Primary analysis 36
8.3. Secondary analysis of the primary endpoint 36
8.4. Analysis of secondary endpoints 37
8.5. Analysis Populations 37
8.6. Imputation 37
8.7. Analysis of Adverse Events and Severe Adverse Events 38
8.8. Interim Analysis 38
9. Safety 39
9.1. Definition of Adverse Events and Side Effects 39
9.1.1. Adverse Events 39
9.1.2. Adverse Drug Reaction 39
9.1.3. Unexpected Adverse Drug Reaction 39
9.1.4. Serious Adverse Event and Serous Adverse Reaction 40
9.2. Period of Observation 40
9.3. Documentation and Reporting of Adverse Events 40
9.3.1. Documentation and Reporting of Adverse Events by the Investigator 40
9.3.2. Assessment of Severity and Causality 41
9.3.3. Outcome of the Adverse Reactions 42
9.3.4. Sponsors Assessment of the SAEs 42
9.3.5. Follow-up of Initial Report 43
9.3.6. Suspected Unexpected Serious Adverse Reaction (SUSAR) 43
9.3.7. Expedited Reporting to the Regulatory Authorities 43
9.3.8. Unblinding of the Medical Product 44
9.3.9. Examination and Report of Changes in the Risk to Benefit Ratio 44
9.3.10. Reporting to Data and Safety Monitoring Board 44
9.3.11. Report to the Investigator 44
9.4. Annual Safety Report 45
10. Regulatory Consideration 46
10.1. Ethical Conduct of Clinical Study 46
10.1.1. Good Clinical Practice, Declaration of Helsinki and legal Provision 46
10.2. Subject Information and Informed Consent 46
10.3. Insurance 46
10.4. Confidentiality 47
10.5. Responsibility of the Investigator 48
10.6. Registration of the Trial 48
10.7. Continuous Information to Independent Ethics Committee 49
10.8. Approval of Protocol and Subsequent Amendments 49
11. Publications 50
11.1. Reports 50

11.2. Publication 50
12. Financing 51
13. References 52
14. Appendix 53
14.1. The Tisdale Score 53

II.a) List of Tables

Table 1: Study Timelines 21

II.b) List of Figures

Figure 1: Frequency and Scope of the Study Visits 20

III. Signature Page

The present trial protocol was subject to critical review and has been approved in the present version by the persons signed.

Sponsor: The Universitätsklinikum Tübingen is sponsor for the purpose of § 4 (24) German Medicinal Products Act with complementary regulations. The internal responsibility to comply with the obligations of the sponsor in terms of these regulations stays with Prof. Dr. med. Wolfgang Bethge.

Date: ___________

Signature:____

Name: Prof. Dr. med. Wolfgang Bethge

Function: Delegated sponsor and person in charge to meet the obligations of the sponsor

Date: ___________

Signature:____

Name: Prof. Michael Bitzer

Function: Coordinating Investigator (LKP)

Date: ___________

Signature:____

Name: Prof. Peter Martus

Function: Biometrician

Declaration of the Principal Investigator

By my signature, I agree to supervise personally the conduct of this study at my study site and to ensure its conduct is in compliance with the protocol, informed consent, the national laws, the ICH Good Clinical Practices Guidelines and the Declaration of Helsinki. I will train the involved personal accordingly.

Date: ___________ Signature:_________________________________

Name (block letters):__________________________
Function: Principal Investigator

Date: ___________ Signature:
____

Name (block letters):_______________________________
Function: Deputy Principal Investigator

Address of the Study Center: __________________________________

____

____

IV. Contacts

**Sponsor**	Universitätsklinikum Tübingen<br>Geissweg 3<br>72076 Tübingen
**Principal Investigator**	Prof. Dr. med. Michael Bitzer<br>Tübingen Universitätsklinikum<br>Medizinische Klinik<br>Otfried Müller Str. 10<br>72076 Tübingen
**Co-Principal Investigator**	Prof. Dr. med. Wolfgang Bethge<br>Dr. med. Siri Göpel<br>Prof. Dr. Peter Kremsner<br>Prof. Dr. Benjamin Mordmüller
**Investigators**	Prof. Dr. med. Nisar Malek<br>Prof. Dr. med. Stefanie Joos<br>Dr. med. Florian Kreth
**Collaborators**	Prof. Dr. Thomas Iftner<br>Prof. Dr. Oliver Kohlbacher
**University hospital Ulm**	Prof. Dr. med. Thomas Seufferlein<br>PD Dr. med. Beate Grüner<br>Dr. med. Lukas Perkhofer<br>Dr. med. Thomas Ettrich
**Biometry and data management**	Prof. Peter Martus und Dr. Christoph Meisner<br>Institut für Klinische Epidemiologie und angewandte Biometrie<br>Silcherstr. 5<br>72076 Tübingen<br>E-Mail: peter.martus@med.uni-tuebingen.de
**Project management**	Dr. Manola Zago<br>ZKS Tübingen<br>Frondsbergstraße 23<br>72070 Tübingen<br>Tel. +49 7071 29 85629<br>Email: zks-pm@med.uni-tuebingen.de

Monitoring

ZKS Tübingen
Frondsbergstraße 23
72070 Tübingen
Tel. +49 7071 29 85635
Email: zks-mo@med.uni-tuebingen.de

SAE-Management

Zentrum für Klinische Studien
Frondsbergstr. 23
72070 Tübingen
phone: +49 7071 2985545
e-mail: zks-pv@med.uni-tuebingen.de

SAE reporting

Fax : +49 7071 29 25205

V. Abbreviations

ADR	Adverse Drug Reaction
AE	Adverse Event
AMG	German Medicinal Products Act (Deutsches Arzneimittelgesetz)
BfArM	Bundesinstitut für Arzneimittel und Medizinprodukte
BMBF	Bundesministerium für Bildung und Forschung
CRF	Case Report Form
CTCAE	Common Toxicity Criteria for Adverse Events
DBL	Data Base Lock
DSMB	Data and Safety Monitoring Board
EC	Ethics Committee
ECG	Electrocardiogram
FSI	First Subject In
GCP	Good Clinical Practice
GCP-V	Good Clinical Practice Ordinance (GCP-Verordnung)
IC	Informed Consent
ICH	International Conference on Harmonization of Technical Requirements for Registration of Pharmaceuticals for Human Use
IIT	Investigator Initiated Trial
IMP	Investigational Medicinal Product
IMPD	Investigational Medicinal Product Dossier
ISF	Investigator Site File
LSI	Last Subject In
LSO	Last Subject Out
SAE	Serious Adverse Event
SmPC	Summary of Product Characteristics (deutsch: Fachinformation)
SDV	Source Data Verification
SUSAR	Suspected Unexpected Serious Adverse Reaction
TMF	Trial Master File

VI. Synopsis

Sponsor	Universitätsklinikum Tübingen
Title	Randomized controlled trial of hydroxychloroquine versus placebo in early ambulatory diagnosis and treatment of elderly COVID19 patients
Short Title	Test and treat COVID 65 plus
EudraCT-Number	2020-001482-37
ClinicalTrials.gov-Number	Not yet applied
Internal Study Code	
Coordinating Investigator	Prof. Dr. med. Michael Bitzer
Study Design	Randomized placebo controlled double blind multicentric Phase II/III trial
Trial Sites	University Hospitals Tuebingen and Ulm, Germany
Number of Patients	120 (including 10% drop outs) in the first stage of adaptive design, approximately 300 to 400 patients in the second stage
Patient Population	Adults older than 64 years, i.e. 65 and above
Length of study/ Time Lines	Total trial duration: approximately 8 months<br>Duration for individual patient: Study treatment: 7 days<br>Follow-up: 2 months<br>FSI (First Subject In): Q2/2020<br>LSI (Last Subject In): Q4/2020<br>LSO (Last Subject Out): Q1/2021<br>DBL (Data Base Lock): Q2/2021<br>Statistical Analyses Completed: Q2//2021<br>Trial Report Completed: Q3//2021
Aim of the Study	The aim of this trial is to identify an effect of ambulatory treatment in early COVID19 disease with hydroxychloroquine on the rate of hospitalization or death in elderly patients

Objectives/Endpoints	**Primary efficacy endpoint:**<br>● Rate of hospitalization or death at day 7 after study inclusion<br>**Exploratory endpoints**:<br>● Duration of hospitalization<br>● Time to hospitalization<br>● Reason for hospitalization<br>● Severity of disease at hospitalization being classified by SpO2%, Respiratory rate, Blood pressure, pulse, mental state, need of oxygen supply<br>● All-cause mortality within 30/60 days<br>● COVID19 related mortality within 30/60 days<br>● Proportion requiring invasive ventilation<br>● Proportion admitted to ICU<br>● Rate of viral clearance defined as SARS-CoV2 specific RNA copy number <100 in throat swabs on day 7<br>● Optional rate of viral clearance on d 14, 21<br>● Hydroxychloroquine levels on day 7, optional at hospital admission

Inclusion Criteria	1. Written informed consent<br>2. Age ≥ 65 years<br>3. Mild to moderate symptomatic respiratory tract Infection defined as not requiring hospital admission, SpO2 >94%, respiratory rate <20, mental state alert, no signs of septic shock<br>4. Proven SARS-Cov2 infection by throat swab (PCR)<br>5. Onset of symptoms within the last 3 days before randomization<br>6. Must be able to adhere to the study visit schedule and other protocol requirements in the investigator’s opinion. I.e. must be able to answer to questions concerning symptoms and side effects and must be able to consent to the informed consent.<br>7. Tisdale score (see appendix) ≤ 6

Exclusion Criteria	1. Hospitalization at study inclusion<br>2. Weight <50 kg<br>3. Acute myocardial infarction or Angina pectoris<br>4. Severe heart failure, characterized as NYHA class 3 or 4<br>5. Use of concomitant medications that prolong the QT/QTc interval.<br>6. QTc >450ms<br>7. Bilirubin ≥ 1,5 x UNL, (except for known M. Meulengracht)<br>8. AST/ALT ≥ 3 x ULN<br>9. Albumine ≤ 2.8 g/dl<br>10. Hemoglobin ≤ 9 g/dl<br>11. Leucocytes ≤ 2000/µl<br>12. Neutrophiles ≤ 1000/µl<br>13. Thrombocytes ≤ 100.000/µl<br>14. Troponin elevation<br>15. BNP > 500 pg/ml<br>16. Creatine kinase > 5 x ULN<br>17. Creatinine >1,5 mg/dl<br>18. Uncorrected hypopotassemia or hypomagnesemia<br>19. History of hypoglycemic events<br>20. History of or present cardial arrhythmia (except atrial fibrillation or paroxysmal supraventricular tachycardia)<br>21. Bradycardia <60 beats/min<br>22. Psoriasis<br>23. Myasthenia gravias<br>24. Epilepsy<br>25. Immunodeficiency syndromes or need for highly immunosuppressive medication<br>26. Pre-existing medication with hydroxychloroquine<br>27. Known G6PD deficiency<br>28. Participation in another interventional study<br>29. Known hypersensibility to hydroxychloroquine and its derivates

Statistics, Safety Variables and Stopping Rules	**Rate of Hospitalization or death:** Hospitalization or death within 7 days after study inclusion<br>**Description of the primary efficacy analysis and population:** Intention to treat analysis in patients with baseline data. Multiple imputation for dropouts. Chi-square test stratified for study center in both stages of an adaptive design (details section 8)<br>**Safety**: descriptive<br>**Exploratory endpoints:** non confirmatory (details section 8)<br>**Sample size:** 2:1 Randomization, 36 Placebo, 72 Verum + increase for 10% drop outs = 120 pts in total.<br>First stage of adaptive design, approximately n = 300-400 in total
Study Intervention/ Study Medication	**Experimental intervention:** Hydroxychloroquine sulfate<br>**Control intervention:** Placebo
Description of the Investigational Medicinal Product	**Hydroxychloroquine sulfate** (HCQ) is a drug used for the prevention and treatment of protozoal infections and auto-immune diseases.<br>Formulation: Encapsulated tablet, 200 mg hydroxychloroquine sulfate (155 mg hydroxychloroquine base)<br>Administration: oral<br>**Placebo**<br>Formulation: Encapsulated tablet<br>Administration: oral
GCP-compliance	The Investigator and the Sponsor will ensure that this study is conducted in full conformity with relevant regulations and with the ICH guidelines for GCP E6(R2) 2016 and the German Medicines Law.
Sites	This is national study with 2 or more sites in Germany

1. Introduction

In the face of the pandemic spread of COVID19, health care systems are facing major challenges due to large amounts of patients requiring hospital care and intensive medicine care. As soon as the capacities of the individual health care system are surpassed, optimized care for all cannot be guaranteed any more.
Containment strategies in Germany include quarantine of infected persons and quarantine of contact persons for 14 days (incubation period). On a population level reduction of contacts has been pursued by most affected countries with various measures such as closure of schools, shops, gastronomy and in the extreme a total lockdown.
Fatality and severe courses of the disease are more common in the older population above the age of 60 years (WHO 2020).
30% of patients in Wuhan were in the age group from 60 to >80 years, but 80% of the fatalities occurred in these age groups (CCDC 2020)
Outbreaks in longterm care facilities have been observed in several countries, posing specific challenges in containment and isolation within the facility, thereby affecting and threatening the most vulnerable. Lack of personal protective equipment enhances the problem.

Treatment options include best supportive care and management of ARDS and sepsis (WHO 01/2020). A large scale of potential antiviral drug candidates has been defined partly by molecular analyses, partly by in vitro testing or known effects against structural similar MERS and SARS (Guangdi Li et al 2020).

In vitro data show that hydroxychloroquine can inhibit SARS-CoV-2 replication (Wang et.al 2020) and preliminary results from small numbers of COVID-19 patients in China and France (Gao et al. 2020 and Gautret et al. 2020) suggest that chloroquine enhances viral clearance. Published literature until now reports mainly non-controlled use of the medication in hospitalized patients. Median time from onset of symptoms to hospitalization in a large Chinese cohort was 7 days (CCDC 2020), so it can be hypothesized that treatment is delayed substantially especially in the population most at risk for severe course of disease if treatment is only implemented in hospitalized patients.

We propose to conduct a placebo-controlled trial in elderly COVID-19 patients with mild to moderate disease in an outpatient setting in Germany to assess the effect of hydroxychloroquine treatment on the rate of hospitalization. Secondary endpoints will explore time to hospital admission, safety and tolerability of hydroxychloroquine, clinical outcome including rate of ICU admission, rate of mechanical ventilation and viral clearance in vivo as well as longterm mortality of all causes after 60 days.
Many other drugs are under investigation for repurposing them in the treatment of COVID19. This trial will set up a structure to investigate hydroxychloroquine versus placebo as the now most promising candidate.

1.1. Trial Rationale / Justification

Chloroquine or Hydroxychloroquine are widely prescribed drugs for the treatment and prophylaxis of malaria or autoimmune disease. They have a known safety profile, which allows their use under known precautions in a large population. Use in the above mentioned indications is usually done over several months (Michalski et al. 2010).

In vitro results revealed several mechanisms that promote the use of Chloroquine and Hydroxychloroquine in Sars-CoV2-Infection:

Zhou et al. (2020) demonstrated that SARS-CoV-2 uses angiotensin converting enzyme 2 receptor (ACE2) as a cellular entry receptor.

Especially in this cellular entry mechanism, Sars-CoV2 has strong structural homologies to SARS-CoV (Xu et al. 2020) which has already been studied for several years. Vincent et al (2005) showed that Hydroxychloroquine blocks the terminal glycosylation of the receptor, resulting in inhibition of SARS-CoV infection and it affects the activation of p38 mitogen-activated protein kinase (MAPK), involved in the replication of HCoV-229E (Kono et.al. 2008).

Furthermore, Hydroxychloroquine increases the endosomal pH, thus inhibiting infection through SARS-CoV2 since virus to cell fusion requires a low endosomal pH (Wang et al 2020). In vitro, Hydroxychloqoruine was able to block viral replication at an EC of 1.1µM (Wang et al.2020)

Several clinical trials have been initiated and are currently being conducted to confirm in vivo efficacy of Hydroxychloroquine or Chloroquine. Gao et al (2020) reported superiority of Chloroquine compared to Placebo in more than 100 patients both in terms of clinical outcome and reduction of viral load.

Chinese expert consensus recommend that patients diagnosed as mild, moderate and severe cases of COVID-19 pneumonia and without contraindications to chloroquine, be treated with 500 mg chloroquine twice a day for ten days. However, until now the above mentioned study has not been published, reporting is limited to a short abstract.

Following these findings, a open-label, non-randomized clinical trial was conducted in France by Gautret et al (March 2020) on a small cohort of 36 COVID19 patients. Treatment was Hydroxychloroquine 3x200mg/ day for 10 days. They showed a significant effect on viral clearance in the treatment group (20 Patients) compared to non-treatment group (16 Patients) at day 6.

Many clinical trials are ongoing in several countries (China, France, USA, Germany) using chloroquine or hydroxychloroquine for the treatment of COVID19 disease caused by SARS-CoV2.
These studies have focused on the treatment of hospitalized patients. In other studies median time from symptom onset to hospitalization was 7 days (Wang et al 2020), thus potential treatment might be delayed in a considerable amount of patients.
Early diagnosis by testing in an early stage of disease and early intervention by effective drugs might alter the course of the disease especially in the population most at risk for severe course of disease.
In a population-based approach, altering the course of disease in this population most at risk can prevent hospital admissions and intensive care thus alleviating the pressure on the healthcare system.
In the proposed study patients will be randomized in a 2:1 ratio to either hydroxychloroquine or placebo.
Patients and investigators, as well as treating physicians will be blinded to treatment-allocation.

1.2. Benefit / Risk Assessment

COVID-19 constitutes a severe threat to public health as no treatment or preventive medical intervention is available. In patients suffering from severe COVID-19 only supportive care and treatment of complication is available. Prevention is only feasible by social distancing and isolation of positive patients.
Screening of known drugs with antiviral properties has revealed possible candidates on a molecular level and in vitro results have further enhanced possible treatment options.
The last data released by Gautret et al. in March 2020 showed a small cohort of 36 COVID19 patients. Treatment was Hydroxychloroquine 3x200mg/day for 10 days. They showed a significant effect on viral clearance in the treatment group (20 Patients) compared to non-treatment group (16 Patients) at day 6. Mean Hydroxychloroquine concentration was 46µg/ml (N20).

In this study, hydroxychloroquine will be assessed against placebo in a specific at risk population at a very early stage of disease. HCQ will be used since in vitro and in vivo studies show it to be better tolerated and have higher anti-SARS-CoV-2-activities in in vitro assays (Xuetin Yao et al 2020).

In some countries, the above mentioned candidate drugs (including Hydroxychloroquine) are already being used in clinical treatment, however, data of randomized clinical controlled trials are scarce. This caused the EMA to issue a warning of the use of Hydroxychloroquine outside controlled clinical trials at the end of March 2020 and the WHO highly encouraged conducting controlled clinical trials.
As for now, the optimal dosing regimen in the context of COVID19 remains unclear. Existing study protocols use dosages ranging from 200mg/day to 600mg/day, some of them initiated with a slightly higher loading dose (CDC 03/2020).

A recent study (Yao X et al 2020) compares achievable plasma levels and estimated lung trough concentrations in different dosing regimens with the in vitro activity of hydroxychloroquine against SARS-CoV2 and proposes a regimen with 800mg loading dose followed by 5 days 400mg/day. However, these data rely on plasma level estimations from patients ranging from 20-50years and are not correlated to clinical outcome.

Based on these results we propose for patients over 64 years a reduced loading dose of 600mg on the first day followed with 400mg/day divided in 2x200mg for 6 more days resulting in a total duration of therapy of 7 days. Measurement of Hydroxychloroquine-levels will be performed on day 7, the blood sample being collected and frozen at -20°C to avoid unblinding.

Associated risks are mostly related to toxicity of HCQ. In the above mentioned dosing scheme, a cumulative dose of 3 g will be taken. At this dose, gastrointestinal symptoms, headaches, sight problems and psychological symptoms are common (≥1/100). Described serious adverse events include retinopathy (particularly with

cumulative dosed >200g), hypoglycaemia, QT-interval prolongation, cardiomyopathy, neurological and psychological syndromes.

Hydroxychloroquine has been used as a longterm treatment in several rheumatological disorders and shortterm treatment of Malaria in doses similar to the above mentioned with known sideeffects and known riskfactors. This allows a safe usage under known precautions:

Exclusion criteria of the study protocoll reflect known side effects and interactions of Hydroxychloroquine and thus allow the usage with minimized risk.

On the other hand fatality and severe courses of COVID19-disease are more common in the older population above the age of 60 years (WHO 2020). 30% of patients in Wuhan were in the age group from 60 to >80 years, but 80% of the fatalities occurred in these age groups (CCDC 2020). Hence an effective antiviral therapy wold be highly beneficial especially to the population included in the study.

In this trial, testing and study inclusion will be done in the outpatient setting. A follow-up by video or telephone conference will be performed to observe drug intake and collect adverse events during treatment phase on a daily base on working days and once during the weekend (i.e. 6 out of 7 days). After treatment phase follow-up by telephone calls will be done on day 10, 30, 60 (+/- 2 days). Additionally an App will be offered to patients to document their symptoms in a standardized way in terms of a symptom diary.
ECG and blood sampling will be done prior to randomisation to allow exclusion of patients at special risks.
After seven days patients clinical status will be assessed at the study center including physical examination, blood sample, ECG and a second throat swab. If feasible throat swabs will be also collected on day 14 and 21.

1.3. Advisory Committee

Data and Safety Monitoring Board (DSMB):
An independent Data and Safety Monitoring Board (DSMB) will be assembled. The DSMB will be composed of independent experts in the field of infectious diseases and an independent statistician, assessing the progress, safety data and critical efficacy endpoints. The mission of the DSMB will be to ensure the ethical conduct of the trial and to protect the safety interests of patients in this trial.
The DSMB will receive a report listing summarizing all the relevant safety data every two weeks from the inclusion of the first patient, after the inclusion of 20 patients in the verum group a safety analysis will be performed not embedded in the framework of the adaptive design according to Bauer and Köhne (1994).The analysis will be provided to the DSMB. The DSMB will provide the sponsor with recommendations regarding trial modification, continuation or termination of the trial. The DSMB will then be informed every 30 days.
After treatment of 36 Placebo und 72 Verum Patients (+ increase for 10% drop outs = 120 patients in total) the primary endpoint will be analysed. The outcome will be

discussed with the DSMB. The members of the DSMB, their responsibilities as well as the frequency and format of meetings and communication procedures are described in further details in a DSMB Charta filed in the Trial Master File (TMF).

An emergency meeting of the DSMB may be called at any time should questions of patient safety arise and necessary safety reports will be provided. Meetings may be convened as conference calls as well as in person.

2. Study Objectives

2.1. Primary Objective and Endpoint

- Rate of hospitalization or death at day 7 after study inclusion

2.2. Exploratory Objectives and Endpoints

- Duration of hospitalization
- Time to hospitalization
- Reason for hospitalization
- Severity of disease at hospitalization
  being classified by SpO2%, Respiratory rate, blood pressure, pulse, mental state, need of oxygen supply
- All-cause mortality within 30/60 days
- COVID19 related mortality within 30/60 days
- Proportion requiring invasive ventilation
- Proportion admitted to ICU
- Rate of viral clearance defined as SARS-CoV2 specific RNA copy number <100 in throat swabs on day 7
- Optional rate of viral clearance on day 14, 21
- Hydroxychloroquine level on day 7

3. Study Design

The study is a randomized placebo controlled multicentric Phase II/III trial. The study scheme is shown below in Figure 1 (Frequency and Scope of the study visits). Details on the adaptive statistical design can be found in section 8.

Each patient will be given a first dose of 600 mg IMP or the equivalent number of placebo capsules (3 capsules) at the day of inclusion. From the 2nd day on, each patient will get 200 mg or the equivalent number of placebo capsules twice a day (400mg/day) until day 7 (6 more does of 400 mg); a cumulative dose of 3 g.

D0/D1	D 2-6	D7 +1	D 10 +/-2	D14 +/-1	D21 +/-1	D30 +/-2	D60 +/- 2	
• Recruitment<br>• Throat swab before Inclusion<br>• Inclusion<br>• Blood sample<br>• ECG<br>• Questionnaire<br>• Clinical examination	Telephone Follow-up and Treatment Compliance	Blood sample<br>ECG<br>Throat swab<br>Clinical examination	Telephone follow-up	Throat swab (optional)	Throat swab (optional)	Telephone follow up	Telephone Follow-up	
**Treatment**								
	• **Questionnaire for symptom assessment via Telephone on all working days, once during the weekend during treatment (d2-6), optional additionally via App**<br>• **Primary Endpoint reached at day 7**<br>• **Further samples (Throat swabs, stool, blood) might be collected on these days for study associated research purposes. Blood for research purposes will be limited to 40ml in total.**							

Figure 1: Frequency and Scope of the Study Visits

3.1. Study Duration and Schedule

The duration of the trial for each subject is expected to be 2 months. The duration for each individual subject includes 7 days study treatment and 2 months follow-up time after randomization (see also Figure 1).

The overall duration of the trial is expected to be approximately 8 months including preparatory phase. Recruitment of subjects will start in April 2020. The actual overall duration or recruitment may vary. The study timelines are described in Table 1.

Table 1: Study Timelines

Total trial duration	8 months
Duration for in individual patient	Study treatment: 7 days<br>Follow-up: 2 months
FSI (First Subject In)	Q2/2020
LSI (Last Subject In)	Q4/2020
LSO (Last Subject Out)	Q1/2021
DBL (Data Base Lock)	Q2/2021
Statistical Analyses Completed	Q2/2021
Trial Report Completed	Q3/2021

3.2. End of Study

The end of the study is defined as the last visit of the last patient.

4. Study Population

4.1. General Criteria for Subject Selection

Adult male and female ≥65 years of age patients with positive COVID-19 diagnosis and fulfilling the below outlined inclusion criteria will be enrolled into the study.
Trial population will consist of both genders. Gender distribution in the trial is supposed to reflect the distribution in the real patient's population, there will be no prior defined quantitative ratio between females and males.

4.1.1. Inclusion Criteria

Subjects meeting all of the following criteria will be considered for admission to the trial:

1. Written informed consent
2. Age ≥ 65 years
3. Mild to moderate symptomatic respiratory tract Infection defined as not requiring hospital admission: SpO_2 >94%, respiratory rate <20, mental state alert, no signs of septic shock
4. Proven SARS-Cov2 infection by throat swab (PCR)
5. Onset of symptoms within the last 3 days before randomization
6. Must be able to adhere to the study visit schedule and other protocol requirements in the investigator's opinion. i.e. must be able to answer to questions concerning symptoms and side effects and must be able to consent to the informed consent.
7. Tisdale score (see appendix) ≤ 6

4.1.2. Exclusion Criteria

1. Hospitalization at study inclusion
2. Weight <50 kg
3. Acute myocardial infarction
4. Severe heart failure classified as NYHA class 3 or 4
5. Use of concomitant medications that prolong the QT/QTc interval.
6. QTc >450ms
7. Bilirubin ≥ 1,5 x UNL (except for known M. Meulengracht)
8. AST/ALT ≥ 3 x ULN
9. Albumine ≤ 2.8 g/dl
10. Hemoglobin ≤ 9 g/dl
11. Leucocytes ≤ 2000/µl

12. Neutrophiles ≤ 1000/µl
13. Thrombocytes ≤ 100.000/µl
14. Troponin elevation
15. BNP > 500 pg/ml
16. Creatine kinase > 5 x ULN
17. Creatinine >1,5 mg/dl
18. Uncorrected hypopotassemia or hypomagnesemia or hypocalcemia
19. History of hypoglycemic events
20. History of or present cardial arrhythmia (except atrial fibrillation or paroxysmal supraventricular tachycardia)
21. Bradycardia <60/min
22. Psoriasis
23. Myasthenia gravis
24. Epilepsy
25. Immunodeficiency syndromes or need for highly immunosuppressive medication
26. Pre-existing medication with hydroxychloroquine
27. Known G6PD deficiency.
28. Participation in another interventional study
29. Known hypersensibility to hydroxychloroquine and its derivates

5. Information on the Investigational Medical Product

Investigational medicinal product (IMP):
Name of IMP: Hydroxychloroquine
Pharmaceutical formulation: film-coated tablets in capsule

Dosage: 200 mg

Route of administration: oral

Storage conditions: no specific storage conditions

Manufacturer: Ratiopharm GmbH (encapsulated by the Klinikapotheke, Universitätsklinikum Ulm)

Comparator: Placebo, encapsulated by the Klinikapotheke, Universitätsklinikum Ulm

Comparator: Placebo

Route of administration: oral

5.1. Manufacturing of the Investigational Medicinal Product

The IMPs will be obtained commercially and encapsulated by the Klinikapotheke, Universitätsklinikum Ulm

5.2. Labeling of the Investigational Medicinal Product

The labels for the IMP and Placebo will contain information to meet the applicable regulatory requirements. Samples of the labels have neen approved by the German Authority and are filed in the trial master file (TMF).

5.3. Storage of the Investigational Medicinal Product

All trial medication must be kept in a locked area with access restricted to designated trial staff at ≤25°C. The manufacturer has no specific storage instructions.

5.4. Drug Accountability, Therapy Compliance and Disposal

The site investigator will keep an account of the trial medication and acknowledge the receipt of all shipments of the trial medication.
Trial medication will be dispensed to the subject by the investigator. The investigator will document the date of dispensary, subject identification, batch/ serial numbers or other identification of trial medication. The investigator will also keep accurate records of the quantities of trial medication dispensed and used by each subject. Details will be recorded in the Case Report Form (CRF). The site monitor will periodically check the supplies of trial medication held by the investigator to ensure the correct accountability of all trial medication used. At the end of the trial, all unused trial medication will be destroyed by the investigator after approval of the sponsor.

5.5. Method of Treatment Assignment

All consenting adult patients having confirmed COVID-19 will be recruited and randomly and blindly allocated in a 2:1 ratio to either IMP or placebo.

5.6. Dose Schedule

Each patient will be given a first dose of 600 mg IMP or the equivalent number of placebo capsules (3 capsules) at the day of inclusion. From the 2nd day on, each patient will get 200 mg or the equivalent number of placebo capsules twice a day (400mg/day) until day 7 (6 more does of 400 mg).

6. Study Procedures and Examination Method

This Study will consist of the following consecutive phases: Study entry, treatment and follow-up. Time-points and trial procedures are shown on Figure 1.

6.1. Study Entry

6.1.1. Patient's Informed Consent

The subject has to be informed both in writing and verbally by the investigator before any study-specific procedure is performed. Each patient will be informed about the modalities of the clinical study in accordance with the provided patient information. The patient is given sufficient time to consider participation in the clinical trials and ask for additional advise if needed. Informed consent from the patient will be obtained using a form approved by the responsible ethical committee (EC). The patient and informing investigator must each personally date and sign the informed consent form with an integrated declaration on data privacy protection on the same day. The original signed documents will be part of the investigator's site file and retained with it and a copy included the insurance policy of the trial will be handed to the patient. The informed consent process is documented in the patient records. Patients who fulfill all the inclusion criteria and none of the exclusion criteria will be eligible to participate in the trial. Only after the signature of the informed consent the patient will undergo study-procedures and will be randomized.

6.1.2. Screening and Enrollment

The patients will be screened for eligibility, informed about the trial and consented for participation immediately after diagnosis of COVID-19. Next the patient will be randomized and the treatment will be allocated. For screening the following examinations are necessary:

- 12 channel ECG
- Baseline cardiac enzymes (CK, Troponine, BNP)
- Weight, height (cm), pulse, blood pressure, body temperature (°C), respiratory rate and SpO2
- Baseline Laboratory including differential blood count, Hb, creatinine, calculated creatinine clearance, electrolytes (K, Na, Mg, Ca), glucose, troponine, CK, BNP, Bilirubine, AST, ALT, CRP, PCT, LDH, albumin, D-Dimeres, HbA1c

6.1.3. Randomisation

The biostatistical center will produce a randomization list (block randomization) with varying block length and stratified for study center. This list is provided for packaging to the pharmceutical unit.

6.1.4. Blinding

IMP will be given to the patients orally. To mask the IMP versus placebo the original tablets will be encapsulated. After this step IMP and placebo will not be distinguishable. Only the pharmaceutical unit is aware of group allocation according to the randomization list. Breaking the code shall be handled conservatively and, if possible, following consultation of the investigator and the sponsor.

6.1.4.1. Unblinding

Breaking the code will be done in case that patients' safety is compromised and a causal link to the IMP is suspected when allocation status is not known. This can be an emergency situation that is potentially related to IMP administration or the occurrence of an SAE where information on allocation status is important for proper clinical management. Unblinding can be considered in case of hospital admission if the above mentioned circumstances are met.

The study is unblinded to clinical investigators and participants following database lock.

For emergency unblinding events, a copy of sealed envelopes of the safety monitor or the delegated study team member can be used. Envelopes will be provided by the biostatistical center.

6.1.5. Concomitant Medication and Treatments

Relevant additional medications and treatments administered to the subjects on entry to the trial or at any time during the trial are regarded as concomitant medications and treatments and must be documented on the appropriate pages of the CRF.

Concomitant medication with a known risk of QT-Intervall prolongation must not be used. This includes especially antibiotics such as Macrolides or Azithromycin.

Preexisting chronic medication with these drugs will not allow inclusion into the study.

Information on QT-prolonging medication is available at https://crediblemeds.org/index.php/drugsearch.

All patients will receive the standard of care in the participating centres. While patients will not be allowed to participate in other studies on experimental substances, it is at the discretion of the treating physician to administer any treatment that is available and he/she deems beneficial.

6.2. Treatment Phase

After randomization and treatment allocation the patients will receive the full course of the study drug or placebo. The patients will then take the first dose of the allocated treatment under observation.

Patients will be monitored via telephone or videoconference regularly. An additional monitoring will be offered via an App to prove feasibility in this study context. The app will function as a medical diary to document symptoms. Other functions especially diagnostic tests, allocation of the carrier or diagnostic analysis are not intended. Data from the app will be integrated in the study-database automatically by pseudonomized datatransfer. The patients informed consent will contain a

specifically dedicated section to consent to the datatransfer. All data entered into the app is stored centrally on AWS servers in Frankfurt. The transport of data between the app and the database is encrypted with Perfect Forward Secrecy. The software libraries used are certified according to FIPS-140-2 level 1. In addition, the data stored locally on the mobile phones as well as the central database are fully encrypted.

The app does not require any permissions (except for using the camera to scan the QR code to register the study). Furthermore, no background information such as device IDs, address lists or location data is collected. Only a randomly assigned pseudonym (12 digits with mixed letters and numbers), the answers to the health questions and the ID assigned in the study are stored and transmitted. No further information is stored or processed.
Monitoring will be done on all working days and once during the weekend from day 2-7 and on day 10 (+/-2), 30 (+/-2), 60 (+/-2). Throat swabs will be performed compulsory on day 0 and 7 (+1), if feasible additionally on day 14 (+/-1) and 21 (+/-1). Clinical controls will be performed by a physician or study-nurse. Visit procedures will include symptom assessment by questionnaire and clinical examination. Blood draw for assessment of full blood count and routine clinical chemistry will be performed on days 0, 7, and according to medical necessity. ECG and measurement of cardiac enzymes will be performed day 0, and on day 7. In case of a prolongation of the QTc_time > 500 ms or an increase of > 60ms comparted to the value at baseline, the patient will be referred to a specialist in cardiology and will spend at least one night under observation. The cardiologist could decide that the treatment of the patient has to be unblinded.The patient continues as study patient the follow-up within the study

Further samples (Throat/Buccal swabs, sputum, stool, urine, rectal swab, blood) might be collected on these days for research purposes. Blood for research purposes will be limited to 40 ml in total and will require no additional puncture but will be taken only with the planned sample.
If feasible, a blood sample to determine the levels of Hydroxychloroquine will be derived on day 7 or on hospital admission to allow for assessment of dosing accuracy. In case of medical necessity of unblinding, the analysis can be initiated immediately after officially unblinding the patient. For all other cases, the blood sample will be taken and stored at -20°C. Analysis will be done at interim analysis of the subjects included or after 60 days.

Daily intake of the medication after day 0 will be done by the patient and also followed up by videoconference/phone call/App.

6.3. Assessment of Efficacy

The efficacy will be assessed by clinical course, endpoint being rate of hospitalization or death at day 7 after study inclusion. Throat swabs will provide further information on rate of viral clearance combined with clinical assessment.

Viral Diagnostics:

The test used to define SARS-COV-2 positivity as inclusion criterium has to be based on a dual target real time PCR assay (E/S and or R gene) and should be CE or IVD marked, but can be also for RUO (research use only) if it has a proven sensitivity to detect at least $2x10^3$ copies viral RNA (LOD = level of detection) per naso- or oropharyngeal swab. Cross-reactivity to other coronaviruses besides SARS and MERS needs to be excluded. All tests must be run with a positive control and an internal control to identify possible RT-PCR inhibition and to confirm the integrity of the reagents of the test components. Diagnostic tests need to be performed in a biosafety lab level 2 and handling of the material needs to be done in a laminar flow hood by persons that wear a personaly safety equipment consisting of protective eye-wear, FFP2 masks and additional protective gowns covering the lab coat. Gloves need to be worn in a way that excludes contact of the skin with potential infectious material. Ananlysis of the results should be made by personel trained in diagnostic virology

6.4. Assessment of Safety

Safety will be assessed on each visit by the study physician until the endpoint is reached and at all subsequent scheduled visits and contacts as well as at any unscheduled visit. This assessment can also be made by telephone visits, if direct contact to patient is not needed (e.g. no sampling is required).

6.5. Premature termination of clinical trial for a trial subject

Reasons for premature termination of trial for an individual trial subject are:

1. Death
2. Withdrawal of consent
3. Patient lost to follow-up
4. Major protocol violation
5. If, in the investigator’s opinion, continuation of the trial would be detrimental to the subject’s well-being
6. QTc_time prolongation > 500 ms or an increase of > 60ms comparted to the value at baseline on ECG at day 7 the patient immediately discontinues HCQ as per protocol. The patient will be referred to a specialist in cardiology and will spend at least one night under observation. The cardiologist could decide that the treatment of the patient has to be unblinded. The patient continues as study patient the follow-up within the study
7. Noncompliance

The investigator decides about withdrawal of subjects from trial treatment in case of occurrence of criteria mentioned above. In all cases, the reason for withdrawal must be recorded in the CRF and in the subject's medical records. In case of withdrawal of a subject at his/ her own request, the reason should be determined and documented.

All examinations scheduled for the last trial day will be performed and documented as far as possible, subject to the consent of the patient. These subjects will enter the regular follow-up of the trial, unless the subject has withdrawn his/her consent to any further study-related procedure. If a subject may / will be withdrawn from all trial-related procedures (including follow-up visits) (e.g. at his/her own request), this will not result in any disadvantages for the patient.

All ongoing Adverse Events (AEs)/ Serious Adverse Events (SAEs) of withdrawn subjects have to be followed-up until no more signs and symptoms are verifiable or the subject is on stable condition.

Premature termination should be avoided. In case of a premature termination of therapy, reasons/circumstances and if applicable the final status have to be documented. If the patient does not withdraw the consent for further follow-up, he/she should be followed-up as planned.

6.6. Premature closure of a trial site

Premature closure of a trial site has to be considered if:

- The conduct of the study is not compliant with the protocol or the legal regulations, or
- The data quality is not sufficient

The premature closure of a site will be decided by the sponsor.

Site principal investigators may terminate his/her participation in the study. If this occurs they should provide a written statement of the reasons for terminating participation and should provide the sponsor with all available and up-to-date study data.

The sponsor may also decide to terminate participation of an investigator or study center for the following reasons:

- Breach of agreement
- Serious non-compliance to protocol or the legal regulations
- Insufficient patient recruitment

If a participating center closes, or is closed, prior to termination of the whole trial, the sponsor expects that data from patients already entered into the trial will be reported as per protocol. Details on further treatment and follow-up of patients on study have to be discussed with the site principal investigator.

6.7. Premature termination of the trial

The trial may be prematurely terminated, if in the opinion of the sponsor and coordinating investigator there is sufficient reasonable cause. Written notification documenting the reason for study termination will be provided to the investigators.
In case of the following situations, a premature termination of the trial has to be considered:

- Serious adverse drug reactions / not justifiable toxicity
- Substantial changes in risk-benefit considerations
- New insights from other trials
- Insufficient efficacy
- Insufficient recruitment rate

The DSMB will monitor the study conduct and the safety aspects of the trial on a regular basis, and will give recommendations to the coordinating investigator/ the sponsor whether to stop the trial or to change the trial protocol. The sponsor will then decide on the actions to be taken. According to the German Medicinal Products Act (§42a), the trial may be suspended or prematurely terminated by decision of the competent authority (BfArM).

6.8. Follow Up

The patients will be followed up until 2 months after randomization. During this time safety assessment will be performed and at all follow up visits samples will be collected of exploratory endpoints analysis.

6.9. End of Study for Subjects

The end of Study for a subject enrolled in this trial is defined as the last study visit. (Last Follow-up visit).

6.9.1. Plan for Treatment or Care after End of Study

After end of the Study for a subject enrolled in this trial no additional treatment or care is required except for normal routine care.

7. Quality control and Quality assurance

7.1. Risk-based approach

During protocol development, processes and data that are critical to ensure human subject protection and the reliability of trial results were identified.

The identified risks were evaluated against existing risk controls by considering:

- The likelihood of errors occurring
- The extent to which such errors would be detectable
- The impact of such errors on human subject protection and reliability of trial results.

In case of unacceptable risks, risk reduction activities were defined and incorporated e.g. in the protocol, monitoring plan and agreements.

Results will be communicated to those who are involved in or affected by such activities.

The sponsor periodically reviews risk control measures to ascertain whether the implemented activities remain effective and relevant, considering emerging knowledge and experience.

7.2. Monitoring

Monitoring for this study is provided by the Zentrum für Klinische Studien Tübingen (ZKS Tübingen). The monitoring will be conducted according to ZKS Tübingen internal Standard Operating Procedures (SOPs) and a dedicated monitoring manual for the study. The monitoring timelines include, for all centres, initiation visit, regular monitor visits during the course of the trial as well as a close out visit. Usually, Monitoring will end with the last visit after full documentation of the last patient enrolled (close out visit). All investigators agree that the monitors regularly visit the trial site, assure that the monitors will receive appropriate support in their activities and will have access to all trial-related documents.

The aims of the monitoring visits are as follows:

- Check informed consent documents
- Monitor trial subject safety (occurrence and documentation/reporting of Serious Adverse Events (SAEs) and Adverse Events (AEs)).
- Check completeness and accuracy of entries on the CRFs.
- Validate entries on the CRFs against those in the source documents (source data verification (SDV)).
- Check the Drug Account
- Evaluate compliance with the trial protocol
- Assess whether the trial is being performed according to GCP at the trial site

- Discuss with the investigator aspects of trial conduct and any deficiencies found
- A monitoring visit report is prepared for each visit describing the progress of the clinical trial and any problems

7.3. Audits/ Inspections

In addition to the monitoring activities, audits can be conducted by the sponsor or assigned auditors. These audits may include checking the whole course of the study, documentation, trial centre, investigators and the monitor.

The competent regulatory authorities may also conduct inspections.

With his/her participation in the study, the investigator agrees to support the activities of the auditor/inspector, provide her/him with direct access to the source documents, study documentation and give her/him the opportunity to audit/inspect the study site, laboratory facilities, storage of the investigational product, etc.

7.4. Documentation: Collection, Handling, Storage and Archiving of Data

7.4.1. Case Report Form

The trial Case Report Form (CRF) is the primary data collection instrument for the trial. All data requested on the CRF must be recorded. All missing data must be explained.

For this project, electronic Case Report Forms (eCRFs) will be used.

The correctness of entries in CRFs will be confirmed by of an authorized investigator. The investigator is responsible for ensuring that all sections of the CRF are completed correctly and that entries can be verified against source data. The investigator has to verify the eCRFs via electronic signature after completion of the CRFs.

7.4.2. Source Data

Source data is all information, original records of clinical findings, observations or other activities in a clinical trial necessary for the reconstruction and evaluation of the trial. Source data are contained in source documents. Examples of these original documents and data records include: hospital records, clinical and office charts, laboratory notes, memoranda, patients' diaries or evaluation checklists, pharmacy dispensing records, recorded data from automated instruments, copies or transcriptions certified after verification as being accurate and complete, x-rays, CTs, MRIs, ultrasound reports, patient files, and records kept at the pharmacy, at the laboratories, and at medico-technical departments involved in the clinical trial.

7.4.3. Data Handling

Data will be entered in a validated GCP conform database or in a paper-based CRF until the database is available. After completion of data entry, checks for plausibility, consistency, and completeness of the data will be performed. Based on these checks, queries will be produced.

7.4.4. Storage and Archiving of Data

According to the EU Clinical Trial Regulation 536/2014 all essential trial documents (e.g. CRF) will be archived for at least 25 years after the trial termination. The investigator(s) will archive all trial data (source data and Investigator Site File (ISF) including subject identification list and relevant correspondence) according to the Guideline ICH GCP (E6).

8. Statistical Analyses

Before unblinding of the trial, a statistical analysis plan (SAP) will detail the analysis of the trial.

8.1. Study Population Definition

Details of participants screened meeting the study inclusion criteria and participating baseline visit, those who are eligible and randomized, those who are eligible but not randomized, those who withdraw from the study after randomization and those who are lost to follow-up will be summarized in CONSORT flow diagrams.
The number of participants discontinuing from the study will be tabulated by reason for study discontinuation. The number (%) of participants attending scheduled follow-up visits will be reported. The baseline value is defined as the last available value before randomization.
Once enrolled, all participants are randomized into two treatment groups. Participants in each group will be described with respect to demographic and baseline characteristics.
Intention to Treat (ITT) population: all recruited participants that received at least one dose of the study drug.
Per Protocol (PP) population: all participants having received a full course of the study medication and not withdrawn due to protocol violations until the respective follow-up duration.

8.1.1. Sample Size and Power Consideration

Fixed design

From data presented by the CDC (MMWR 2020) for march 12th until march 16th hospitalization rates in the USA were between 28.6% and 43.5%% in the age group between 65-74 ys and between 30.5% and 58.7% in the age group of 75-84 ys. We have to expect considerably smaller rates in our study population, without clear prior evidence. Thus, as a primary guess, we assume a hospitalization rate of 25% in the Placebo group.
Taking into account possible adverse drug reactions in the group with Hydroxychloroquine a relevant absolute risk reduction of 15% (number needed to treat: 7) in this group would lead to a hospitalization rate of 10%. In a fixed sample size design and taking into account the 2:1 relation of Chloroquine vs. Placebo a total of 213 evaluable patients (142 vs. 71) would be necessary to achieve a power of 80%, and a total of 291 (194 vs. 97) evaluable patients would be necessary to achieve a power of 90%: Taking into account the short time interval of seven days between randomization and assessment of the primary endpoint, a drop out rate of at most 10% seems to be realistic. Thus, to achieve a power of 80%, 237 patients should be recruited, for a power of 90%, 324 patients have to be recruited. We chose the power of 90% and thus the fixed sample size would be n=326 patients (including one degree of freedom for center effects). Note, however, that we will use an

adaptive design which will enable us to adjust the sample size after an interim analysis (see below).

Adaptive Design according to Bauer and Köhne
In view of the weak evidence of the Placebo and Verum hospitalization rates, and the short time from randomization to the assessment of the primary endpoint, an adaptive design is adequate. The primary endpoint will be analysed in Step 1 if 108 patients (72 vs. 36) with primary endpoint are observed. However, all subjects with baseline information will be included according to the intention-to-treat principle. The interim analyses will be conducted by a statistician not involved in the design and analysis of this trial to preserve blinding of the study statistician. Based on the p-value p_1 of this stage and the observed effect, the statistical planning of the second stage, including the p-value p_2 necessary to obtain an overall significant result will be performed. The analysis will be done for $\alpha_0 = 0.4$ (stopping for futility after stage 1), $\alpha_1 = 0.0115$ (stopping for success after stage 1), and $c_\alpha = 0.00380$ (critical value of the product p_1 times p_2 after stage 2) for a one-sided overall α of 0.025 in the terminology of Bauer and Köhne (1994). This leads to p-values of 0.0115 in the first stage and 0.00380/p1 in the second stage necessary to get a significant overall result. The sample size of 108 patients with primary outcome available in stage one leads to the following probabilities: If we assume a true effect of 0.25 vs. 0.10, the power to proceed to the second stage or stop early for success is 0.98. In case of a true effect of 0.20 vs. 0.10 the power is still 0.90. The power for early stopping for success (which is not intended in this situation) is 0.42 (0.25 vs. 0.10) and 0.22 (=.20 vs. 0.10). If we reversely assume, that there is a harm of Chloroquine of 25% vs. 10% (20% vs. 10%) hospitalization, the probability to erroneously proceed to stage 2 is less than 1% (5%).

8.2. Primary analysis

Statistical analysis of the primary outcome will be a chi-square test stratified for study center (Mantel Haenszel) with type 1 error 0.025 (one-sided). This will be done identical in stage 1 and stage 2 of the adaptive design. One-sided p-values can be obtained by using the signed square root of the chi-square value.

8.3. Secondary analysis of the primary endpoint

In secondary analyses of the primary enpoint, multiple logistic regression models will be used by including terms for age, gender, duration of symptoms, and baseline virus load as covariates additional to study arm and treatment center. No subgroup analyses are planned, however, if significant interactions between one of these variables and the treatment arm are observed, effect modification will be assessed by chosing two subgroups, using the median in case of continuous covariates.

8.4. Analysis of secondary endpoints

Analysis of secondary outcomes will be done according to scaling and observed distributions of the data. Concretely, the following methods will be applied:

- Duration of hospitalization (median, quartiles, Mann-Whitney test)
- Time to hospitalization (90%, 75%,median time, Kaplan-Meier method with log rank test)
- Reason for hospitalization (95% confidence limits of rates and their differences, Chi-Square test and)
- Severity of disease at hospitalization (median, quartiles, Mann-Whitney test)
- All-cause mortality within 30/60 days (99%, 95%, 90%, Kaplan-Meier method with log rank test)
- COVID19 related mortality within 30/60 days (99%, 95%, 90%, Kaplan-Meier method with log rank test)
- Proportion requiring invasive ventilation (95% confidence limits of rates and their differences, Chi-Square test)
- Proportion admitted to ICU (95% confidence limits of rates and their differences, Chi-Square test)
- Rate of viral clearance defined as SARS-CoV2 specific RNA copy number <100 in throat swabs on day 7 (95% confidence limits of rates and their differences, Chi-Square test)
- Optional rate of viral clearance on d 14, 21 (95% confidence limits of rates and their differences, Chi-Square test)

Even so p-values will be reported, the analyses should be considered non confirmatory. Additional exploratory analyses might be performed using adequate multiple regression models (linear, logistic, Cox proportional hazard model).

8.5. Analysis Populations

The primary analysis population is the intent to treat population. Only patients with baseline assessment will be included in this population to make imputation of missing values for the outcome feasible.

8.6. Imputation

We will use a multiple imputation approach for drop outs using the baseline data. These will include age, gender, duration of symptoms as well as virus load at baseline. Dependent on the observed missing pattern, data after baseline and before day seven might be included in the imputation model. Data are assumed as missing at random (MAR). A monotone pattern over time will be used, if data fit to this assumption.

8.7. Analysis of Adverse Events and Severe Adverse Events

The following summary statistics on AEs will be presented by treatment group:

- Number and percentage of participants with any AE during the study period
- Number and percentage of participants with any SAE during the study period
- Number and percentage of AEs which caused early discontinuation of the investigational medical product (IMP)

All participants in the treatment group are considered in the denominator unless otherwise specified. A descriptive overview of all AEs will be reported cumulatively. In addition, AEs will be reported, only considering the most severe event for all participants with multiple occurrences of events. In the final report a full listing of AEs will be provided.

8.8. Interim Analysis

See section adaptive designs. Additionally, a safety monitoring of 20 patients in the verum group will be performed not embedded in the framework of the adaptive design according to Bauer and Köhne (1994)

9. Safety

9.1. Definition of Adverse Events and Side Effects

9.1.1. Adverse Events

Any untoward medical occurrence in a patient or clinical investigation subject administered a pharmaceutical product and which does not necessarily have a causal relationship with this treatment. An adverse event (AE) can therefore be any unfavorable and unintended sign (including an abnormal laboratory finding), symptom, or disease temporally associated with the use of a medicinal (investigational) product, whether or not related to the medicinal (investigational) product.

An AE may be:

- New symptoms/ medical conditions
- New diagnosis
- Changes of laboratory parameters
- Diseases and medical consequences of an accident
- Worsening of medical conditions/ diseases existing before clinical trial start
- Recurrence of disease
- Increase of frequency or intensity of episodical diseases

A pre-existing disease or symptom will not be considered an adverse event unless there will be an untoward change in its intensity, frequency or quality. This change will be documented by the investigator.

In general, abnormal laboratory findings or clinical events without clinical significance (based on the investigator´s judgement) should not be recorded as AEs.

Surgical procedures themselves are not AEs; they are therapeutic measures for conditions that require surgery. The condition for which the surgery is required may be an AE. Planned surgical measures permitted by the clinical trial protocol and the condition(s) leading to these measures are not AEs, if the condition leading to the measure was present prior to inclusion into the trial.

AEs are classified as "non-serious" or "serious".

9.1.2. Adverse Drug Reaction

Adverse reaction means all untoward and unintended responses to an investigational medicinal product unrelated to the dose administered.

9.1.3. Unexpected Adverse Drug Reaction

An unexpected Adverse Drug Reaction (ADR) is a reaction which nature or severity is not consistent with the applicable product information available for the IMP. Expected ADRs are listed in the appropriate reference document: Summary of Product Characteristics (SmPC [Fachinformation in Germany]).

9.1.4. Serious Adverse Event and Serous Adverse Reaction

AEs are classified as "non-serious" or "serious".

A serious adverse event (SAE) is one that at any dose:

- Results in death.
- Is life-threatening (the term life-threatening refers to an event in which the subject was at risk of death at the time of event and not to an event which hypothetically might have cause death if it was more severe).
- Requires subject hospitalization or prolongation of existing hospitalization.
- Results in persistent or significant disability/ incapacity.
- A congenital anomaly / birth defect.

In this Study the following events have to be reported as SAE

- any arrhythmia that needs treatment
- any life threatening cardiac conduction disorder

9.2. Period of Observation

For the purpose of this trial, the period of observation for collection of adverse events for each patient goes from the time of first administration of the IMP until 30 days after first dose. AEs will be elicited at all follow up visits as well. SAE will be documented during the whole trial period.
All adverse events that occur in the course of a clinical trial regardless of the causal relationship must be monitored and followed up until the outcome is known or no more information is achievable.

9.3. Documentation and Reporting of Adverse Events

9.3.1. Documentation and Reporting of Adverse Events by the Investigator

The investigator must document all adverse events that occur during the observation period set in this protocol on the pages provided in the case report form. Additional instructions may be provided in the investigator file and in the case report form itself. The following approach will be taken for documentation:
All adverse events (whether serious or non-serious) must be documented on the “adverse event” page of the case report form.
If the adverse event is serious, the investigator must complete, in addition to the “adverse event” page in the case report form, a “serious adverse event report form” at the time when the serious adverse event is detected. The investigator will

document the date when he/she or any employee was first aware of the report and fax all SAE reports (initial and follow-up reports), even if they are incomplete, directly or latest 24 hours upon receipt to the representative of the Sponsor:

ZKS
SAE reporting Fax +49 7071 29 25205
E-mail: zks-pv@med.uni-tuebingen.de

The investigator should also assess the severity and the causal relationship between the event and the trial medication.

9.3.2. Assessment of Severity and Causality

The investigator will also provide an assessment of the severity of the event according to CTCAE criteria (Version 5.0) and causal relationship between the event and each of the investigational products or trial procedures.
AEs and SAEs should be evaluated for severity according to the following scale:

- Grade 1 - mild event: Causing no limitations of usual activities; the patient may experience slight discomfort.
- Grade 2 - moderate event: Causing some limitation of usual activities; the patient may experience annoying discomfort.
- Grade 3 - severe event: Causing inability to carry out usual activities; the patient may experience intolerable discomfort or pain.
- Grade 4 - life threatening or disabling event
- Grade 5 - death related to event

The investigator should also assess the causal relationship between the event and the trial medication to following scale:
Is there a reasonable causal relationship that the AE is related to the study medication?
Y (Yes) There is a reasonable possibility that the IMP/s caused the AE.
N (No) There is no reasonable possibility that the IMP/s caused the AE and other causes are more probable.
If no, other possible causes have to be specified.

9.3.3. Outcome of the Adverse Reactions

The information about the outcome of an AE at the time of the last observation should also be reported. The following classification will apply:

**Recovered/ Resolved:**	All signs and symptoms of an AE disappeared without any sequels at the time of the last interrogation.
**Recovering/ resolving:**	The intensity of signs and symptoms has been diminishing and/ or their clinical pattern has been changing up to the time of the last interrogation in a way typical for its resolution.
**Not recovered/ not resolved:**	Signs and symptoms of an AE are mostly unchanged at the time of the last interrogation.
**Recovered/ resolved with sequel:**	Actual signs and symptoms of an AE disappeared but there are sequels related to the AE.
**Fatal**	Resulting in death. If there are more than one AE only the adverse event leading to death (possibly related) will be characterized as ‘fatal‘.
**Unknown**	The outcome is unknown or implausible and the information cannot be supplemented or verified.

9.3.4. Sponsors Assessment of the SAEs

All SAE will be subject to a second assessment by the trial Sponsor or authorized second assessors.

The second assessor will fill out a ‘Second Assessment Form’ for each SAE containing.

- Event serious yes/no
- Relationship between SAE and IMP
- Expectedness of SAE according to the reference document: SmPC of Hydroxychloroquine.
- Benefit / risk assessment for the trial regarding change as a result of SAE.

9.3.5. Follow-up of Initial Report

Information not available at the time of the initial report (e.g. end date for the adverse event or laboratory values received after the report) must be documented on a “Serious Adverse Event” form with the box “Follow-up” checked under “Report type”.

All patients who have adverse events, whether considered associated with the use of the investigational products or not, must be monitored to determine the outcome as far as possible. The clinical course of the adverse event will be followed up according to accepted standards of medical practice even after the end of the period of observation, until a satisfactory explanation is found or the investigator considers it medically justifiable to terminate follow-up. Should the adverse event result in death, a full pathologist’s report should be supplied, if possible.

The sponsor will identify missing information for each SAE report and will require follow up information in regular intervals from the investigators until all queries are resolved or no further information can be reasonably expected. All responses to queries and supply of additional information by the investigator should follow the same reporting route and timelines as the initial report.

9.3.6. Suspected Unexpected Serious Adverse Reaction (SUSAR)

SAEs have to be assessed by the second assessor whether they are both suspected, i.e. related to IMP and ‘unexpected’, i.e. the nature and / or severity of which is not consistent with the applicable product information. They are then to be classified as Suspected Unexpected Serious Adverse Reactions (SUSARs).

In case, either the investigator or the second assessor classifies the SAE as related to IMP and the SAE is unexpected as assessed by the second assessor it will be categorized as a SUSAR.

All SUSARs are subject to an expedited reporting to the responsible ethics committee(s), the competent higher federal authority (i.e. BfArM) and to all participating investigators.

9.3.7. Expedited Reporting to the Regulatory Authorities

In this study any death, any arrhythmia that needs treatment and any life threatening cardiac conduct disorder must be evaluated by the DSMB and are subjected to expedite reporting to the authority and the ethic-commission.

Fatal and life-threatening SUSARs

The competent authority and the ethics committee responsible must be informed by the Sponsor of all fatal or life-threatening SUSARs. This must be done immediately, at the latest seven calendar days after becoming aware of the minimum criteria for reporting. In all cases, attempts must be made to obtain further relevant information, which must be supplied to the competent authority and the ethics committee in overall charge within a further eight days. Furthermore, if a trial subject dies, this information must be additionally passed on to the ethics committee responsible for the region in which the death occurred.

SUSARs that are not fatal or life-threatening

The authority and the ethics committee responsible will be informed without delay by the sponsor or CI of all SUSARs, at the latest within 15 calendar days of becoming aware of the minimum criteria for reporting. Further relevant details will be passed on as soon as possible.

If the information at the time of reporting is incomplete, further information to enable adequate assessment of the case will be requested from the reporter or other available sources.

9.3.8. Unblinding of the Medical Product

If it is medically imperative to know which trial medication the subject is receiving, the investigator or authorized person should open the randomization envelope. The investigator or the person who breaks the blind must record the date and the reasons for unblinding in the CRF, in the subject's medical record and on the randomization envelope. Whenever possible, the Sponsor / CI should be contacted before the blind is broken.

9.3.9. Examination and Report of Changes in the Risk to Benefit Ratio

Without delay, and at the latest within 15 days of the decision for the need to do so, the Sponsor / CI will inform the competent authority, the ethics committee responsible of any events or factors that could result in a review of the risk-benefit ratio of the IMP. These consist of especially:

- Individual reports of expected serious ADRs with an unexpected outcome.
- A clinically relevant increase in the rate of occurrence of expected ADRs.
- SUSARs in trial subjects who have already completed the follow-up period of the clinical trial ("end-of-trial visit").
- Factors emerging in connection with trial conduct or the development of the IMP that may affect the safety of persons concerned.

9.3.10. Reporting to Data and Safety Monitoring Board

In this study any death, any arrhythmia that needs treatment and any life threatening cardiac conduct disorder must be evaluated by the DSMB and are subjected to expedite reporting to the authority and the ethic-commission.

The DSMB will be informed of all safety-relevant events by the Sponsor. The frequency of reporting will be described in the DSMB chapter.

9.3.11. Report to the Investigator

The Sponsor will inform investigators of all SUSARs including all relevant further information within the periods set by the authority.

If new information becomes known that is different from the scientific information given to the investigator, all investigators will be informed of this by the sponsor.

9.4. Annual Safety Report

Once a year, the Sponsor will supply a report on the safety of trial subjects with all available relevant information concerning patient safety during the reference period to the competent authorities. This report will also be supplied to the responsible ethics committee.
The annual safety report will be compiled according to the corresponding ICH guideline E2F „Development Safety Update Report – DSUR“. The safety report will cover all IMPs used in this study.

10. Regulatory Consideration

10.1. Ethical Conduct of Clinical Study

10.1.1. Good Clinical Practice, Declaration of Helsinki and legal Provision

The procedures set out in this trial protocol, pertaining to the conduct, evaluation, and documentation of this trial, are designed to ensure that all persons involved in the trial act according to Good Clinical Practice (GCP) and the ethical principles described in the applicable version of the Declaration of Helsinki.

Clinical Trial in accordance with the German Medicinal Products Act (Arzneimittelgesetz, AMG).

10.2. Subject Information and Informed Consent

Each patient will be informed about the modalities of the clinical study in accordance with the provided patient informed consent (IC). The patient is to be informed both in writing and verbally by the investigator before any study-specific procedure is performed. The patient must be given sufficient time to decide whether to participate in this comparative study and to ask questions concerning this trial. It must also be made clear to the patient that he / she can withdraw from the study at any time without giving reasons and that he / she will not be in any way disadvantaged for this. The subject must give consent in writing. The patient and informing physician must each personally date and sign the informed consent form with an integrated declaration on data privacy protection, whereby the physician must not sign before the patient. Original signed documents will be part of the investigator’s file and retained with it. A copy of the signed informed consent document and study insurance policy must be given to the subject. The documents must be in a language understandable to the subject and must specify who informed the subject. The subjects will be informed as soon as possible if new information may influence his/her decision to participate in the trial. The communication of this information should be documented in the patient chart.

10.3. Insurance

Each patient is insured against any health impairment occurring as a result of participation in the study in accordance with the laws and regulations of the “German Arzneimittelgesetz”. The insurance is covered by HDI-Gerling Industrie Versicherung AG, Am Schönenkamp 45, 40599 Düsseldorf, Policy number 57 010311 03013/03052 and valid throughout the conduct of the study for each individual patient. A copy of the insurance policy and conditions are distributed to the patient upon enrollment into the study and the patient is advised to adhere to the conditions of the insurance policy to safeguard a valid patient insurance.
Further the insurance cover exists for accidents of the patients enrolled to the Study:

- on the direct route from home, workplace or school to the place of study;
- on the direct route from the place of study back to one of these places and

- during the stay at the place of study from the time you enter the place of study until you leave.

The insurance cover does not apply if the normal duration of the journey is extended or the journey itself is interrupted by purely private measures. During the stay at the place of study, no insurance cover exists for the duration of the examination or treatment within the scope of the study, provided that claims can be asserted under the probationer insurance.

10.4. Confidentiality

The data obtained in the course of the trial will be treated according to the European General Data Protection Regulation (Datenschutz-Grundverordnung; DS-GVO) and the applicable local data protection regulations as well as the AMG.
Subjects have to be informed about data protection in the clinical trial and to consent in writing to collect and process their personalized data as well as to transfer their pseudonymized data. The information has to be transparent, precise, easily accessible and understandable and is written in clear and simple language. The written privacy policy must be approved by the responsible ethics committee.

In order to maintain patient privacy, all data capture records, study drug accountability records, study reports and communications will identify the patient by the assigned patient number. The PI determines which persons are authorized to view personal data, the Patient Identification Log is only accessible to authorized study team members. Access rights to personal data (including pseudonymized data) are available to prevent unauthorized access to the data (both electronically and physically). Electronic systems and files are access-regulated, possibly password-protected. Documents and files are kept in lockable rooms, if necessary, cupboards with access control.

The patient name, initials and the full birth date should never be used in any correspondence with the Sponsor or on the Case Report Forms. The investigator will grant monitor(s) and auditor(s) and/or regulatory authorities direct access to the patient's original medical records for verification of data gathered on the data capture records and to audit the data collection process. Direct access includes examining, analyzing, and verifying any recorded data and reports that are important to the evaluation of the monitoring. The investigator is obliged to inform the patient that his/her trial-related records will be viewed without violating their confidentiality and that the collected information will only be made publicly available to the extent permitted by the applicable laws and regulations. All data will be stored either paper based or electronically in a pseudonymous manner and handled strictly confidential. The investigators are obliged to keep all study data and information confidential and to use those data only in context with the persons involved in the trial conduct. Study material or information developed in this trial must not be available to third parties, except for official representatives of the sponsor or regulatory authorities.

Data will be processed at the study site according to the written safety concept of this institution. Access to the data will be strictly limited to authorized persons. Loss of data is excluded due to extensive back-up procedures. All legal requirements concerning data protection and confidentiality will be respected. All authorized persons are sworn to secrecy.
In the case of withdrawal of consent the stored data collected to this time point will be stored and further used. Data not necessary any longer are deleted immediately.
Collected study data will be stored for at least 25 years after the end of the trial, if there are no other regulatory archiving periods. After archiving has expired, the data will be destructed in a data protection compliant manner.

When processing personal data, the following principles must be observed (pursuant to DS-GVO Article 5 “Principles relating to processing of personal data“):

Personal data shall be:

- processed lawfully, fairly and in a transparent manner in relation to the data subject
- collected for specified, explicit and legitimate purposes and not further processed in a manner that is incompatible with those purposes
- adequate, relevant and limited to what is necessary in relation to the purposes for which they are processed
- accurate and, where necessary, kept up to date
- kept in a form which permits identification of data subjects for no longer than is necessary for the purposes for which the personal data are processed
- processed in a manner that ensures appropriate security of the personal data, including protection against unauthorized or unlawful processing and against accidental loss, destruction or damage, using appropriate technical or organizational measures

10.5. Responsibility of the Investigator

The investigator should ensure that all persons assisting with the trial are adequately informed about the protocol, any amendments to the protocol, the trial treatments, and their trial-related duties and functions.
The investigator should maintain a list of sub investigators and other appropriately qualified persons to whom he or she has delegated significant trial-related duties.

10.6. Registration of the Trial

Prior to the beginning of the clinical phase (First Patient In) the Sponsor will register the trial in the EudraCT as well as ClinicalTrials.gov Database.

10.7. Continuous Information to Independent Ethics Committee

According to the German Drug Law (AMG) and the GCP Ordinance, the EC and the competent authority will be informed of all suspected serious unexpected adverse reactions (SUSARs). Both institutions will be informed in case the risk/ benefit assessment did change or any others new and significant hazards for subjects' safety or welfare did occur. Furthermore, a report on all observed SAEs will be submitted once a year – Annual Safety Report.
The EC and the regulatory authorities must be informed of the end of the trial. They will be provided with a summary of trial results within one year after the end of clinical phase.

10.8. Approval of Protocol and Subsequent Amendments

Before the start of the trial, the trial protocol, informed consent document, and any other appropriate documents will be submitted to the independent Ethics Committee (EC) as well as to the competent authority (BfArM). A written favourable vote of the EC and an (implicit) approval by the competent higher federal authority as well as the notification of the local authorities (acc. to §67 AMG) are a prerequisite for initiation of this clinical trial. Before the first subject is enrolled in the trial, all ethical and legal requirements must be met. All planned substantial changes (see §10, (1) of German GCP-Regulation) will be submitted for approval to EC and the competent authority in writing as protocol amendments.

11. Publications

11.1. Reports

Within one year of the completion of the trial, the competent authority and the ethics committee will be supplied with a summary of the final report on the clinical trial containing the principle results.
All reports to the sponsor will be written in English language. All clinical, analytical and statistical results will be presented in a final clinical trial report (CTR). The outline of this report will accord to the ICH Topic E3.

11.2. Publication

The final results of this study will be presented at scientific meetings and published in a peer reviewed leading medical journal of infectious disease medicine or a leading clinical journal. All publications in result of this study are the responsibility of the principal coordinating investigator and the authorship will reflect the contributions of each collaborating centre. Any publication, abstract or presentation based on patients included in this study must be approved by the coordinating investigator.

12. Financing

Financing of the whole study is done by the Ministry of Social Affairs of Baden-Württemberg.

13. References

General and statistics:

This Template is modified from “S01b_TrialProtocol_Template_EN_V6-03-F.doc” Grass and Weiß and “Muster-Prüfpaln_Version v6-03-F” Grass and Weiß
Medicinal Products Act (Arzneimittelgesetz), published on 12 December 2005 (Federal Law Gazette [BGBl.] Part I p. 3394), last amended by Article 3 of the Law of 18 July 2017 (BGBl. I p. 2757)
Ordinance on the implementation of Good Clinical Practice in the conduct of clinical trials on medicinal products for use in humans (GCP Ordinance - GCP-V), published on 09 August 2004 (Federal Law Gazette (BGBl.) I p. 2081), last amended by Article 8 of the Law of 19. Oktober 2012 (BGBl. I S. 2192)

REGULATION (EU) No 536/2014 OF THE EUROPEAN PARLIAMENT AND OF THE COUNCIL of 16 April 2014 on clinical trials on medicinal products for human use, and repealing Directive 2001/20/EC
ICH Topic E3, Note for Guidance on Structure and Content of Clinical Study Reports (CPMP/ICH/137/95), July 1996
ICH Topic E 6 (R2), Note for Guidance on Good Clinical Practice (CPMP/ICH/135/95), December 2016
ICH Topic E 8, Note for Guidance on General Considerations for Clinical Trials (CPMP/ICH/291/95), March 1998
Clinical Trial Facilitation Group, Recommendations related to contraception and pregnancy testing in clinical trials, 15.09.2014)
EMEA-Guideline On Data Monitoring Committees (EMEA/CHMP/EWP/5872/03 Corr), January 2006

REGULATION (EU) 2016/679 OF THE EUROPEAN PARLIAMENT AND OF THE COUNCIL of 27 April 2016 on the protection of natural persons with regard to the processing of personal data and on the free movement of such data, and repealing Directive 95/46/EC (General Data Protection Regulation)
Bauer P, Kohne K (1994) Evaluation of experiments with adaptive interim analyses. Biometrics:1029-1041.

Lachlin and Foulkes 1986 Biometrics. 1986 Sep;42(3):507-19

COVID19 / SARS-CoV2 - related References in order of citation:

Report. World Health Organization (WHO); 2020 16-24.02.2020
CCDC Weekly / Vol. 2 / No. 8
Clinical management of severe acute respiratory infection when Novel coronavirus (2019-nCoV WHO 01/2020)
Guangdi Li Nature Reviews Drug Discovery **19**, 149-150 (2020)

WANG et al. Cell Res. 2020 Mar;30(3):269-271

Gao et al Biosci Trends. 2020 Mar 16; 14(1): 72-73
Gautret et al. 2020 Antimicrobial Agents 17 March 2020 doi 10.1016

CDC: MMWR Morb Mortal Wkly Rep 2020;69:343-346.
Michalski et al primary care clinics and office practice 37, 4, 767-778 2010

Zhou et al 2020 Mar 28;395(10229):1054-1062

Vincent et al. 2005 Virol J. 2005 Aug 22;2:69

Kono et.al. 2008 Antiviral Res. 2008 Feb;77(2):150-2
Wang et al 2020 JAMA Feb 7. doi: 10.1001/jama.2020.1585
Xueting Yao et al Clinical Infectious Diseases, 2020 Mar 9
CDC: https://www.cdc.gov/coronavirus/2019-ncov/hcp/therapeutic-options.html (Accessed on March 31, 2020)
Yao X et al Clin Infect Dis. 2020 Mar 9

14. Appendix

14.1. The Tisdale Score

Age ≥68 years	No 0	Yes +1
Sex	Male 0	Female +1
Patient on loop diuretic	No 0	Yes +1
Potassium ≤3.5 mEq/L<br>Potassium should be potassium determined closest to EKG timing	No 0	Yes +2
Admission QTc ≥450 msec<br>Auto-calculated by EKG	No 0	Yes +2
Being admitted for acute myocardial infarction	No 0	Yes +2
Being admitted for sepsis	No 0	Yes +3
Being admitted for heart failure	No 0	Yes +3
Number of QTc-prolonging drugs given<br>If receiving ≥2 drugs, patient receives 3 points for 1 QTc-prolonging drug as well as 3 additional points for ≥2	None 0<br>1 QTc-prolonging drug +3<br>≥2 QTc-prolonging drugs +6	
